# The dubious value of cerebrospinal fluid adenosine deaminase measurement for the diagnosis of tuberculous meningitis

**DOI:** 10.1186/s12879-017-2221-3

**Published:** 2017-01-31

**Authors:** Pieter Ekermans, Adriano Dusé, Jaya George

**Affiliations:** 1 Department of Chemical Pathology, University of Witwatersrand and National Health Laboratory Service, Johannesburg, South Africa; 2 Department of Clinical Microbiology and Infectious Diseases, University of Witwatersrand and National Health Laboratory Service, Johannesburg, South Africa; 3 Department of Chemical Pathology and Department of Clinical Microbiology and Infectious Diseases, School of Pathology, Faculty of Health Sciences, University of Witwatersrand and National Health Laboratory Service, 7 York Road, Johannesburg, South Africa

**Keywords:** Tuberculosis, Meningitis, Adenosine deaminase, Giusti, Pre-analytical, Youden

## Abstract

**Background:**

The diagnosis of tuberculous meningitis (TBM) can be extremely difficult in the absence of culture confirmation. Cerebrospinal fluid (CSF) adenosine deaminase (ADA) can potentially assist in this regard, although its current value remains unclear. The literature on the usefulness of CSF ADA in TBM diagnosis is inconsistent, especially from an analytical point of view.

**Methods:**

A retrospective analysis of clinical and laboratory data relating to all CSF ADA requests during 2009 and 2010 in a South African quaternary healthcare setting was performed. A CSF ADA cut-off for TBM diagnosis was calculated using receiver operating characteristic curve analysis. The performance of CSF ADA in different infective and non-infective categories was assessed.

**Results:**

In total, 3548 CSF ADA requests were considered over the 2-year period. Of these, 1490 were for patients for whom both a CSF ADA and a mycobacterial culture were requested. The optimal cut-off was calculated at 2.0 U/L (AUC = 0.86; 95% CI = 0.82 – 0.89; *p*-value < 0.01; sensitivity of 85.9% (95% CI of 77.0 – 92.3) and specificity of 77.7% (95% CI of 75.4 – 79.8%); positive likelihood ratio = 3.85 and negative likelihood ratio = 0.18). At this cut-off 13 TBM cases were missed.

**Conclusion:**

An optimal cut-off for routine use could not be established as too many TBM cases were missed. Specimen integrity, lack of ADA assay standardisation and overlap in performance of the assay in different diagnostic categories affect interpretation.

## Background

The diagnosis of tuberculous meningitis (TBM) in general can be extremely difficult in the absence of culture confirmation. A non-definitive test such as cerebrospinal fluid (CSF) adenosine deaminase (ADA) could potentially assist in the regard, although its current value for the diagnosis of TBM remains unclear.

South Africa is a low to middle income country with a heavy burden of infectious diseases, particularly Human Immunodeficiency virus (HIV) and tuberculosis. As the diagnosis of TBM based on culture confirmation can take weeks, clinicians rely heavily on CSF ADA as a rule-in test, because of its fast turn-around time. However, as indicated in Table 1, the sensitivity and specificity of CSF ADA utilised in the diagnostic work-up of suspected TBM patients cited in the literature vary considerably. One of the reasons for this is the different cut-offs used. Mishra and colleagues reported a cut-off of 5 U/L with a sensitivity of 89% and specificity of 92% [1], whilst others have reported a sensitivity of 66.6% and specificity of 90% using a cut-off of 10 U/L [2] and a sensitivity of 100% and specificity of 99% using a cut-off of 20 U/L [3]. Furthermore, additional confounding factors include, among others: differences in the number of participants/specimens [4, 5], ages and HIV-status(where stated) of participants [1, 3, 6–8] and variable definitions of TBM [1, 3, 9] and selection of control groups [4, 6, 8]. Finally, various methods have been used to measure CSF ADA such as those based on the Giusti method [8, 10] or on non-Giusti methods [6, 11]. Many of the methods used are developed in-house [6, 12] (see Table 1).

**Table 1:** Factors confounding the interpretation of literature on CSF ADA

Factors	Variables			
CSF ADA cut-offs	Study from India [1]	5 U/L	Sensitivity: 89%	Specificity: 92%
	Study from India [2]	10 U/L	Sensitivity: 66.6%	Specificity: 90%
	Study from Finland [3]	20 U/L	Sensitivity: 100%	Specificity: 99%
ADA assay types	Giusti method [8, 10]	Non-Giusti method [6, 11]		In-house method [6, 12]
Total number of participants/specimens	Range: 26 – 417 [4, 5]			
Age of participants	Adults only [3]	Children only [1]		Pooled data for adults and children [6]
Definition of TBM groups	Clinical as well culture evidence was used to group participants [1] Culture-positive cases sometimes was < 10 cases [3, 9]			
Definition of control groups	One infective category: bacterial meningitis only [4]			
	More than one infective category: viral/aseptic meningitis, bacterial meningitis, fungal meningitis [8]			
	Infective and non-infective categories: bacterial meningitis, viral meningitis, hydrocephalus, epilepsy, malignancy, subarachnoid haemorrhage, senile dementia, normal CSF [6]			
HIV status	HIV status is not always specified in the literature [7, 8]			

In view of the limitations described above, the objective of this study was to determine an appropriate CSF ADA cut-off level in a quaternary healthcare setting in South Africa to assist in the diagnosis of TBM.

## Methods

**Fig. 1:**
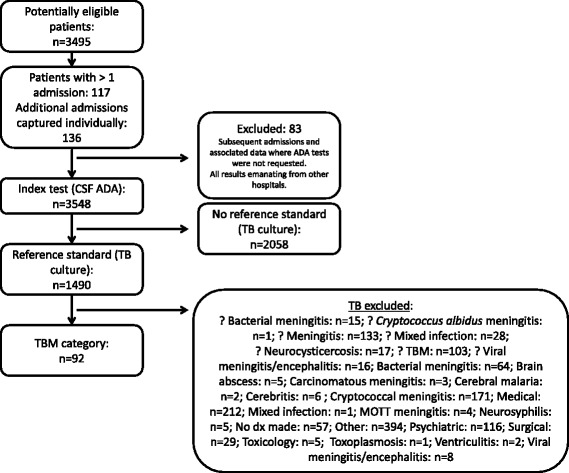
Flow of participants throughout the study. Key to Fig. 1: ? – Suspected infective categories, dx - diagnosis. *Confirmed infective categories*: refers to all those infective categories where the ‘?’ is omitted. For further breakdown of the ‘Confirmed TBM category’ please see Table 2

A retrospective study was conducted over a period of two years (2009 and 2010) in which 3495 medical records were reviewed. As some patients had more than one admission the final number of records eligible for analysis was 3548 (see Fig. 1).

All adult and paediatric, male and female, as well as HIV-positive and HIV-negative patients for whom a CSF ADA was requested were included in this study. If a patient had more than one specimen taken during a particular admission period, only the CSF ADA result from the first specimen was considered. If more than one admission occurred during the study period, each admission was considered as a separate event. All data previously captured on the laboratory information system up to and including the admission data from which the CSF ADA test request originates were considered. Any subsequent admissions and associated data where CSF ADA tests were not requested, as well as results emanating from other hospitals were excluded (see Fig. 1).

The cut-off age of 12 years was arbitrarily applied to separate adult and paediatric patients thus including adolescents in the adult patient category.

For the purpose of this study patients were considered HIV-positive based on one of the following criteria: (1) presence of a positive rapid test as well as a positive enzyme-linked immunosorbent assay (ELISA), (2) presence of a positive initial and confirmatory ELISA, and (3) presence of a positive polymerase chain reaction (PCR) for HIV [13]. In addition (1) the presence of an elevated HIV viral load, and (2) positivity for HIV as stated in the patient file (where tests were done at a different laboratory) were used to assign HIV positivity. In patients aged 18 months or younger, only the HIV PCR result was considered to assign positivity to a patient in this age group [13].

A CD4 absolute cell count of 350 x 10^6^/L was used to further classify patients according to the 2010 World Health Organisation (WHO) guidelines regarding treatment for HIV [14].

**Fig. 2:**
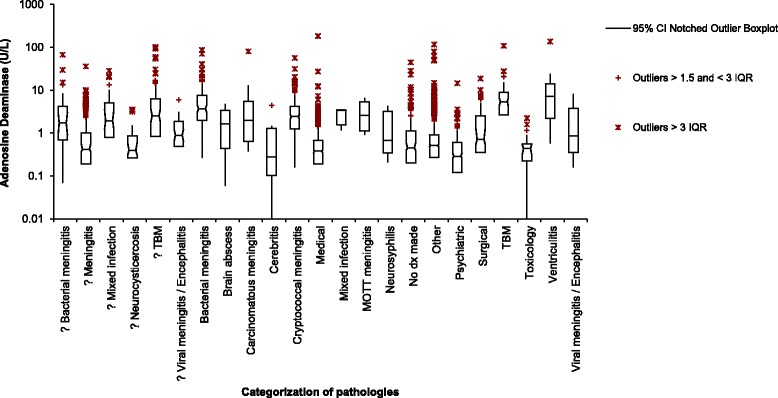
CSF ADA values for each of the categories. Key to Fig. 2: CI - confidence interval; IQR - interquartile range; ? - Suspected infective categories, dx - diagnosis. *Confirmed infective categories*: refers to all those infective categories where the ‘?’ is omitted

Subjects were categorised as ‘Confirmed TBM’ and various infective non-tuberculous pathologies (*confirmed infective categories*) according to definitive evidence (including culture, molecular, and/or serological evidence). For a specific breakdown of the cases considered for the ‘Confirmed TBM’ category, please see Table 2. In addition suspected diagnoses of infective origin were captured separately (*suspected infective categories*) according to the evidence as documented by clinicians in patient files. In cases when CSF ADA was requested as part of a diagnostic work-up, but a non-infectious diagnosis was ultimately made by the clinician, a review of clinical, radiological and histological evidence was performed to exclude the possibility of TBM. The ‘Other’ category includes pathologies not involving CSF or intracranial anatomy. The ‘No diagnosis made’ category includes patients where no clinical, radiological or laboratory evidence was found to assign patients to any of the other categories. CSF ADA performance was assessed in all these categories (see Fig. 2).

**Table 2:** Breakdown of cases considered for the ‘Confirmed TBM’ category (*n* = 92)

Patients		Reason for assigning patients to the ‘Confirmed TBM’ category
88 patients	83 patients	Patients admitted to hospital for the first time and were culture positive
	5 patients	Known patients, where culture positivity was determined in a previous admission during the 2009 – 2010 period; the patient still fell within the 9–12 months treatment period; the CSF specimen in the current admission was negative on culture
4 patients		Patients were positive for acid-fast bacilli on microscopy only^a^

^a^ These four patients where included due to HIV status (including CD4 count < 350 × 10^6^/L) and/or had suspicious radiological findings

The Diazyme ADA assay (Diazyme, United States of America) for the measurement of total ADA was used and performed on a Pentra analyser (Horiba Medical Diagnostics, Japan) with colorimetric detection. The clinical decision limit for TBM used at this healthcare facility was 6 U/L as determined in a South African study by Blake and Berman [6].

The median, interquartile range (IQR), the 95% distribution of values and outliers for the CSF ADA results for each of the categories defined for this study were determined.

All CSF ADA test results for the demographic categories were compared using the Mann–Whitney test for non-parametric data [15]. Similarly CSF ADA results for the ‘Confirmed TBM’ category were compared to all CSF ADA results for patients where culture for *Mycobacterium tuberculosis* complex (TB) on CSF was either not requested or negative, i.e. on the total population for this study (*n* = 3548). Using the Mann–Whitney test, CSF ADA results in the confirmed infective categories were compared between ‘Confirmed viral meningitis/encephalitis’, ‘Confirmed ventriculitis’, ‘Confirmed bacterial meningitis’ and ‘Confirmed TBM’ groups.

Receiver operating characteristic (ROC) curves and the Youden index were generated to establish the optimal cut-off point for ADA interpretation in patients for whom a CSF specimen was submitted for both mycobacterial culture and ADA determination [16]. Using the optimal cut-off point, sensitivity and specificity, as well as positive and negative likelihood ratios were determined. A *p*-value of < 0.05 was considered statistically significant.

The dataset (*n* = 1490) was arbitrarily split into a training and validation cohort after random assignment of values to each of the CSF ADA results to assess for overfitting of the model. The ROC curve and Youden index was generated for the training set. The CSF ADA cut-off point determined was applied to the validation set. The same diagnostic accuracy parameters were determined for the validation cohort as above.

For each of the demographic categories from which a CSF sample was submitted for mycobacterial culture and ADA determination, ROC curve analysis was utilised to determine differences in these groups’ cut-off point for ADA. The area under the curve (AUC) was used to compare the individual ROC curves [15].

CSF sample integrity as determined by its appearance (clear, turbid, bloodstained and xanthochromic) was used to re-categorise CSF ADA results and these categories were compared by establishing the minimum, median, and maximum values as well as the interquartile ranges (IQRs).

A uniform case definition for diagnosis of TBM as determined by Marais and colleagues was used to re-categorise patients and CSF ADA results were assessed in each of the categories [17]. The CSF ADA result is not included in this definition.

Statistical analyses were performed on Analyse-it (Analyse-it Software, Ltd., United Kingdom).

## Results

The majority of the patients in this study were adults (*n* = 3170, 89.3%), of which 2009 individuals (56.6%) were between the ages of 20 and 40 years. Male and female patients were roughly equal in number (*n* = 1721, 48.5% and *n* = 1824, 51.4% respectively). Most patients were HIV-positive (n = 1970, 55.5%). Of the total population, CD4 cell counts for 1456 (41.0%) patients measured ≤ 350 x 10^6^/L. Table 3 summarises the demographic characteristics, HIV status and the CD4 cell count as determined by the medical records reviewed (*n* = 3548).

**Table 3:** Demographic characteristics, HIV status and the CD4 cell count as determined by the medical records reviewed (*n* = 3548)

Age	Adult patients: *n* = 3170 (89.3%)
	Paediatric patients: *n* = 365 (10.3%)
	*No information regarding age available: n = 13 (0.4%)*
Sex	Male patients: *n* = 1721 (48.5%)
	Female patients: *n* = 1824 (51.4%)
	*No information regarding sex available: n = 3 (0.1%)*
HIV status	HIV-positive patients: *n* = 1970 (55.5%)
	HIV-negative patients: *n* = 693 (19.5%)
	*No information regarding HIV status available: n = 885 (25%)*
CD4 cell count	Patients with CD4 cell count > 350 × 10^6^/L: *n* = 235 (6.6%)
	Patients with CD4 cell count ≤ 350 × 10^6^/L: *n* = 1456 (41.0%)
	*No information regarding CD4 cell count available: n = 1857 (52.3%)*

The performance of CSF ADA measurement in each of the infective and non-infective categories is shown in Fig. 2. Figure 2 shows the outlier values in terms of IQR as well as the 95% distribution of the values in each of the categories. Notably the total number of CSF ADA results (*n* = 3548) follow a non-parametric distribution. For the majority of results (*n* = 3415) CSF ADA values were less than 10 U/L. The CSF ADA results in Fig. 2 indicate a considerable overlap, not only in the outliers for each category, but also in each category’s 95% distribution of values. Of note the ‘Confirmed ventriculitis’ category had the widest 95% distribution of values.

In all comparisons of CSF ADA results were higher in adult than paediatric patients (*p* < 0.02), higher in male than female patients (*p* < 0.01), higher in HIV-positive than HIV-negative patients (*p* < 0.01) and higher in patients with CD4 cell counts ≤ 350 × 10^6^/L as opposed to patients with CD4 cell counts > 350 × 10^6^/L (*p* < 0.01).

In Table 4 ‘Confirmed TBM’ CSF ADA results (*n* = 92) are compared to all CSF ADA results for patients where TB culture on CSF was either absent or negative (*n* = 3456). Similar statistically significant results (*p* < 0.01) were obtained from the sub-set analysis of medical records (*n* = 1490) where both culture and ADA was requested on a CSF specimen.

**Table 4:** Statistical comparison of ‘Confirmed TBM’ CSF ADA results with all CSF ADA results for patients where TB culture on CSF was absent and or negative (in U/L)

	TBM (n = 92)	Non-TBM (n = 3456)	
Median	5.4000	0.6000	*p* < 0.01
95% CI	4.1000 – 6.6875	0.6000 – 0.6000	
Ranges	0.0000 - 108.0000	0.0000 - 182.5000	

Key to this table: TBM – refers to ‘Confirmed TBM’ patients; Non-TBM – refers to patients where TB culture on CSF was either not requested or negative

Individually all CSF ADA results in the ‘Confirmed cryptococcal meningitis’, ‘Confirmed bacterial meningitis’, ‘Confirmed viral meningitis/encephalitis’ and ‘Confirmed ventriculitis’ categories were compared with the ‘Confirmed TBM’ category (see Table 5). Statistically significant differences were noted in the comparisons between the ‘Confirmed TBM’ and ‘Confirmed cryptococcal meningitis’ and ‘Confirmed viral meningitis/encephalitis’ categories (*p* < 0.01). This however was not the case in the comparison between the ‘Confirmed bacterial meningitis’ and ‘Confirmed TBM’ categories (*p* = 0.06) and the ‘Confirmed ventriculitis’ and ‘Confirmed TBM’ categories (*p* = 0.42).

**Table 5:** Individual comparison of CSF ADA between different confirmed infective categories and the ‘Confirmed TBM’ category

	‘Confirmed TBM’ (*n* = 92)	‘Confirmed cryptococcal meningitis’ (*n* = 302)	‘Confirmed viral meningitis/encephalitis’ (*n* = 14)	‘Confirmed bacterial meningitis’ (*n* = 134)	‘Confirmed ventriculitis’ (*n* = 16)
Median 95% CI *p*-value	5.4000 4.1000 – 6.6875	2.4000 2.1000 – 2.7000 *p* < 0.01	0.85000 0.3896 – 3.7147 *p* < 0.01	3.7000 3.0176 – 4.4648 *p* = 0.06	7.1500 2.3470 – 13.8145 *p* = 0.42
Ranges	0.0000 – 108.0000	0.2000 – 56.1000	0.2000 – 8.0000	0.3000 – 86.3000	0.6000 – 135.7000

**Fig. 3:**
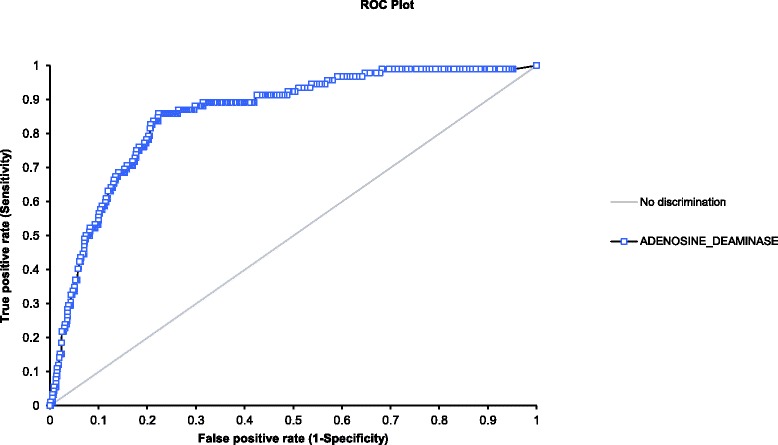
ROC plot for all patients that had both an ADA and a culture for TB requested on a CSF specimen

ROC curve analysis (see Fig. 3) was performed for all events where both an ADA and culture for TB were requested on a CSF specimen (*n* = 1490) and an AUC of 0.86 (95% CI of 0.82 – 0.89, *p* < 0.01) was obtained. This analysis together with the Youden index indicates that the optimal cut-off of CSF ADA in suspected TBM patients is 2.0 U/L with a sensitivity of 85.9% (95% CI of 77.0 – 92.3) and specificity of 77.7% (95% CI of 75.4 – 79.8%). The positive and negative likelihood ratios were 3.85 and 0.18. It is important to note that, even at this cut-off, 13 of 92 ‘Confirmed TBM’ cases (14.1%) were missed (see Table 6). Target and actual sensitivities and specificities at different CSF ADA levels using this ROC curve are noted in Table 7.

**Table 6:** Sensitivity and Specificity of CSF ADA at a cut-off of 2.0 U/L

ADA	2.0 U/L
Youden index	0.636
True positive (n)	79
True negative (n)	1086
False positive (n)	312
False negative (n)	13
Sensitivity (%)	85.9
95% confidence interval	77.0–92.3
Specificity (%)	77.7
95% confidence interval	75.4–79.8
Positive likelihood ratio	3.85
Negative likelihood ratio	0.18

**Table 7:** Target and actual sensitivities and specificities at different CSF ADA levels

Target Sensitivity	First cut-off closest the these targets	Actual Sensitivity	Corresponding Specificity
≥80%	2.3 U/L	81.5%	79.4%
≥85%	2.0 U/L	85.9%	77.7%
≥90%	0.8 U/L	91.3%	57.4%
≥95%	0.5 U/L	95.7%	43%
Target Specificity	First cut-off closest the these targets	Actual Specificity	Corresponding Sensitivity
≥80%	2.4 U/L	80%	78.3%
≥85%	3.3 U/L	85%	68.5%
≥90%	4.5 U/L	90%	55.4%
≥95%	7.3 U/L	95%	33.7%

Training and validation cohorts where determined for the dataset *n* = 1490. The AUC of the ROC curve generated for the training cohort was 0.82 (95% CI of 0.76 – 0.88, *p* < 0.01). For this curve the Youden index determined the appropriate CSF ADA cut-off to be 2.20 U/L. When this cut-off was applied to the validation cohort, the sensitivity, specificity, positive and negative likelihood ratios were 87.5% (95% CI of 74.8 – 95.3%), 77.9% (95% CI of 74.6 – 80.9%), 3.96 and 0.16 respectively.

In sub-set ROC analyses performed for each of the 8 categories listed in Table 3, CSF ADA cut-offs were low (<3 U/L). No optimal cut-off could be determined for paediatric patients and HIV-negative patients with suspected TBM (see Table 8).

**Table 8:** ROC curve results for each of the demographic categories in the population of *n* = 1490

	TBM positive (n)	TBM negative (n)	ROC curve			Sensitivity (%)	Specificity (%)
			AUC	p value	Cut-off (U/L)		
Adult patients	90	1345	0.86	<0.01	2.0/2.2	86.7/85.6	77.8/79.0
Paediatric patients	2	53	0.58	<0.33	0.6	100.0	41.5
Male patients	47	695	0.86	<0.01	2.2 (2.0)	89.4 (89.4)	75.3 (74.1)
Female patients	45	703	0.86	<0.01	2.0	82.2	81.1
HIV positive patients	77	846	0.83	<0.01	2.2 (2.0)	84.4 (85.7)	74.1 (72.8)
HIV negative patients	7	274	0.88	<0.01	0.6	100.0	70.1
Patients with CD4 count ≤ 350 × 10^6^/L	58	653	0.83	<0.01	2.0	86.2	74.4
Patients with CD4 count < 350 × 10^6^/L	4	114	0.93	<0.01	2.7	100.0	86.0

Cut-offs represented was based on the Youden index. In adult patients the Youden index performed similar for cut-off of 2.0 U/L and 2.2 U/L. In brackets performance at a cut-off of 2.0 U/L is indicated

**Fig. 4:**
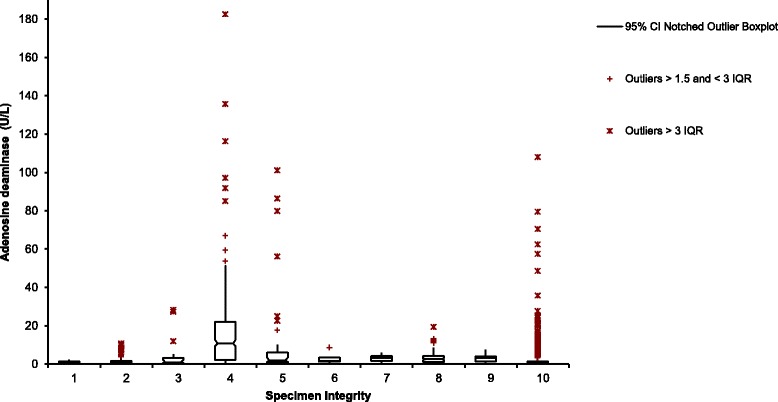
CSF ADA values plotted according to the specimen integrity comments for each sample. The 95% distribution of values (including the median, first and third IQRs) and the outliers are indicated. Key to this Figure: 1 – ungraded bloodstained (*n* = 13), 2 – bloodstained +/− to 2+ (*n* = 196), 3 – bloodstained 3+ to 4+ (*n* = 28), 4 – ungraded xanthochromia (*n* = 81), 5 – xanthochromia +/− to 2+ (*n* = 52), 6 – xanthochromia 3+ to 4+ (*n* = 6), 7 – ungraded turbid (*n* = 11), 8 – turbid +/− to 2+ (*n* = 148), 9 – turbid 3+ to 4+ (*n* = 10), 10 – clear and colourless (*n* = 3003); CI - confidence interval; IQR – interquartile range

All CSF ADA requests (*n* = 3548) were re-categorised according to their integrity and are plotted in Fig. 4. The majority of the specimens were clear and colourless (*n* = 3003, 84.6%). Of note is the considerable spread of CSF ADA results for the ‘Ungraded xanthochromia’ category compared to the ‘Clear and colourless’ category, both in terms of outliers and 95% distribution of values. When excluding all specimens that were not clear and colourless (*n* = 545, 15.4%), no change was noted in the ROC curve analysis and the same cut-off of 2.0 U/L using the Youden index was determined. Furthermore the overlap in CSF ADA results for the different categories remained.

According to the uniform case definition, 68.5% (*n* = 1024) of cases that had both a CSF ADA and a mycobacterial culture requested, fell in the ‘Not TBM’ category. Of these cases, 20.2% (*n* = 207) had CSF ADA results ≥ 2 U/L and 6.2% (*n* = 63) had CSF ADA results ≥ 6 U/L (see Table 9).

**Table 9:** CSF ADA results for the clinical research case definition (in U/L) (*n* = 1490)

Categories	Not tuberculous meningitis (*n* = 1024)	Possible tuberculous meningitis (*n* = 306)	Probable tuberculous meningitis (*n* = 68)	Definite tuberculous meningitis (*n* = 92)
Percentage (%)	68.7	20.5	4.6	6.2
ADA Range	0.0 – 135.7	0.0 – 91.8	0.0 – 6.5	0.0 - 108
Median ADA	0.6	0.9	0.7	5.4
Number of cases in each category ADA ≥ 2	*n* = 207, 20.2%	*n* = 97, 31.7%	*n* = 15, 22.1%	
Number of cases in each category ADA ≥ 6	*n* = 63, 6.2%	*n* = 32, 10.5%	*n* = 4, 5.9%	

## Discussion

TBM is a challenging diagnosis especially in view of the fact that the diagnostic gold standard, culture, generally performs so poorly. We aimed to assess the performance of ADA in all clinical scenarios where CSF sampling was performed and ADA was requested as part of a patient work-up. To date this study is the largest and most comprehensive analysis of ADA performance in CSF samples.

An unusually low cut-off for CSF ADA of 2.0 U/L was determined. This contrasts with the first South African study on the use of CSF ADA that determined a cut-off of 6.0 U/L to indicate probable TBM [6]. In the current study, a similarly low cut-off value (<3 U/L) was determined for all of the categories according to demography, HIV-status and CD4 count. The non-parametric distribution of the CSF ADA results assessed may have contributed to this unusual finding. Selection bias and ascertainment bias may have been introduced in the way the CSF ADA results were chosen. In addition, CSF ADA performance in the ‘Confirmed TBM’ category was compared to a significantly larger set of results in patients where TB culture of CSF was negative (*n* = 1398) and may have skewed the ROC curve analysis. Furthermore, pre-analytical and analytical factors may also have contributed. Similar results were obtained when arbitrarily splitting the dataset (*n* = 1490) in a training cohort and a validation cohort. Overfitting of the model is unlikely to have occurred.

It is important to also note than when confirmed TBM ADA results were compared with all ADA results for patients where TB culture on CSF was either not requested or negative, a statistically significant difference was determined (Table 3). However, the maximum CSF ADA value for the ‘Non-TBM’ category indicated in Table 3 is higher than that for the ‘TBM’ category. In addition, upon detailed comparison of the individual categories, there is significant overlap regarding each category’s 95% distribution of values, as well as their outliers (Fig. 2). This suggests that the CSF ADA result is not reliable for the presumptive diagnosis of TBM. Elevations in non-infectious pathologies have also been noted by other researchers [3, 18].

In clinical practice CSF ADA is used for rule-in purposes. If CSF ADA is used for rule-in/screening purposes the sensitivity should be optimized, but this may result in the cut-off of 2.0 U/L being dropped even further in the current data set. If ADA is used for rule-out purposes the specificity should be optimized, resulting in an elevation of the cut-off with a considerable loss of sensitivity (see Table 7) in the current data set. Given the catastrophic nature of untreated TBM, the consequence of failing to give appropriate therapy in true TBM are considerably worse than the consequences of inadvertently over-treating the disease. The positive likelihood ratio was lower than that published in a recent meta-analysis, whereas the negative likelihood ratio was similar [19]. A positive likelihood ratio of 3.85 does increase the chance of a diagnosis of TBM significantly if there is a high pretest probability of disease. A negative likelihood ratio of 0.18 is of some value if the pretest probability is low as it makes the diagnosis of TBM more than 5-fold less likely. Conversely in all other situations of pretest probabilities, the test is essentially non-contributory. Although these parameters suggest a reasonable performance of CSF ADA, it is of concern that at a cut-off of 2.0 U/L, 13 out of 92 cases of TBM were missed.

When comparing ADA values between prominent infective pathologies (tuberculosis versus other infectious conditions), the absence of a statistically significant difference between the ‘Confirmed bacterial meningitis’ and ‘Confirmed TBM’ categories concurred with findings of a number of studies [18, 20, 21]. Similarly there was no statistically significant difference between the ‘Confirmed ventriculitis’ category and the ‘Confirmed TBM’ category. CSF ADA results were statistically significantly different between the ‘Viral meningitis/encephalitis’ and ‘Confirmed TBM’ categories. This concurred with the research by Donald, et al. [18]. Coovadia, et al. did not find any difference between the 2 categories [21]. A statistically significant difference was found when comparing CSF ADA results between the ‘Confirmed cryptococcal meningitis’ and ‘Confirmed TBM’ categories. Rohani, et al. determined a mean CSF ADA result for TBM cases and those with cryptococcal meningitis as 16.33+/−5.66 U/L and 7.26+/−3.80 U/L respectively. Three of the CSF ADA values for cryptococcal meningitis overlapped with the TBM category in this study [22]. In a country like South Africa, where the HIV seroprevalence is high and both TB and cryptococcal infections are important opportunistic co-morbidities, this phenomenon warrants further investigation.

In the 3548 CSF ADA requests reviewed, results for the categories according to demography, HIV status and CD4 cell count yielded a statistically significant difference for all. Statistically significant differences in ADA results in adult and paediatric patients are in keeping with the work by Donald and colleagues [23]. It has been postulated that a difference in immunological reactivity to tubercular antigen between adult and paediatric patients account for this difference [1]. The reason for the difference in CSF ADA performance between male and female patients is not clear from the literature. It was unexpected to find ADA results to be higher in HIV positive patients and patients with CD4 cell counts less than or equal to 350 × 10^6^/L as intuitively it is assumed that cellular responses are impaired in these patients. Co-morbidities may account for this. Information on treatment for HIV was not collected. It is assumed that if cases were on treatment immune reconstitution would be seen, hence elevated values. Although these differences were statistically significant (*p* < 0.05), in all cases the median CSF ADA values were less than 1.0 U/L.

Several pre-analytical and analytical factors limit adequate interpretation of the results from this study. No specific specimen collection and handling procedures are noted in the Diazyme kit package insert (Diazyme, United States of America) regarding CSF samples. There are no guidelines regarding the stability of ADA specifically in CSF samples. It is unclear what the correct specimen volume should be for optimal assay performance. Feres and colleagues mention in their validation of the Diazyme assay for measurement of ADA (Diazyme, United States of America) in CSF that 22 μL was used [24]. This is different from the 5 μL mentioned in the Diazyme kit package insert (Diazyme, United States of America).

It is important to note the effect of sample integrity on ADA. ‘Ungraded xanthochromia’ accounted for 81 specimens and showed the widest distribution of 95% of their values. Both this category and samples assigned xanthochromia +/− to 2+ (accounting for 52 or 1.5% of samples) showed a wide scatter in their outliers, similar to the category for clear and colourless samples (accounting for 3003 or 84.6% of samples). Ungerer and colleagues demonstrated that erythrocytes contribute 100% of ADA-1 (ADA isoform 1) and polymorphonuclear cells contribute 70% ADA-1 and 30% ADA-1 + CP (two ADA isoform 1 molecules connected by a combining protein) to the total ADA activity in cell extracts [25]. This may explain the performance of CSF ADA in the ‘Confirmed Ventriculitis’ category.

Using a uniform case definition for TBM [17], by weighting the available evidence collected, about two-thirds of cases (*n* = 1024, 68.7%) whom had a CSF ADA and mycobacterial culture requested, fell in the ‘Not TBM’ category. By applying the CSF ADA cut-off of 2.0 U/L, about a fifth of these cases (*n* = 207, 20.2%) would have been treated if the decision to treat was based on the CSF ADA alone. This is about three times more than the number of patients that would have been treated using a cut-off of 6.0 U/L (*n* = 63, 6.2%). Either way, irrespective of whether a cut-off of 2.0U/L or 6.0 U/L is used, a considerable number of patients would have received unnecessary treatment. The uniform case definition was established after extensive literature review and international expert consensus [17]. Although the current study is subject to limitations, the performance of CSF ADA is such that it simply cannot supersede the uniform case definition (see Table 9).

The measurement of ADA in general is complicated by poor standardisation [26]. Giusti and non-Giusti methods have been documented. Most methods are in-house based. The Diazyme assay (Diazyme, United States of America) is a commercially available assay fit for automated platforms. Bota and colleagues produced and certified enzyme reference material for ADA-1 to be used to compare laboratories and verify performance of calibration material and quality control material. This group utilised ADA purified from human erythrocytes. A commutability study was only performed on pleural fluid samples clear of haemolysis and turbidity [27]. The poor standardisation of ADA measurement makes it difficult to interpret the significance of CSF ADA values in the literature. Both the calibrator and control material supplied by Diazyme (Diazyme, United States of America) are bovine serum albumin based. An important question to resolve is how matrix effects interfere with this assay.

No consensus exists regarding the linear range of this assay. The calibration curve for the Diazyme assay (Diazyme, United States of America) lies between 0 – 50 U/L. However, the package insert for the Diazyme assay (Diazyme, United States of America) states that dilution should occur for samples with results > 200 U/L. Delacour, et al., (2010) performed a linearity study and determined the analytical measurement range to be 0.5 – 120 U/L [28].

Ideally control material should reflect analyte levels at which clinical decisions are made. Currently this is true for pericardial, peritoneal and pleural fluids, but not for CSF.

The imprecision of the Diazyme assay (Diazyme, United States of America) at the proposed cut-off of 2.0 U/L is unknown. Data from our validation studies at the lower control level of 10.6 U/L showed a within-run standard deviation of 0.172 U/L, total standard deviation of 0.601 U/L, and the total coefficient of variation of 5.8%. As the allowable coefficient of variation was 7.0%, this performance was deemed to be acceptable. It is possible that the imprecision of the assay is poor at a cut-off of 2.0 U/L. This would require further investigation.

The retrospective analysis of clinical and laboratory data constituted a further limitation of this study. While every effort was made to find all data parameters relevant to the study, this was not always possible due to incomplete data in patient files and on the laboratory information system. This may have affected the categorisation process. Reflex testing of CSF ADA may have occurred. Many of the controls will have had a high pretest clinical probability of other diagnoses. In view of the fact that submission of insufficient CSF volume impacts on the sensitivity of TB culture, it is possible that microbiological confirmation of TBM was missed in some cases, as less than 10% of the 1490 patients who had a culture and ADA requested on a CSF specimen had more than one specimen submitted. As the majority of the population studied were HIV positive and for a quarter the HIV status was not known, these results cannot be extrapolated to non-HIV populations. This requires further investigation.

## Conclusion

The authors cannot recommend CSF ADA as either a rule-in or a rule-out test. A clinical appropriate cut-off could not be determined, as at a cut-off of 2.0 U/L still 13 out of 92 cases of ‘Confirmed TBM’ were missed. Several pre-analytic and analytical factors affect interpretation of CSF ADA results. No definite conclusions can be reached regarding CSF ADA cut-off levels, without fully understanding these factors. The effect of sample integrity cannot be underestimated and there is a pressing need for standardisation of CSF ADA measurement. CSF culture remains the gold standard for the diagnosis of TBM. Its poor performance overall, however, makes it a problematic gold standard to which CSF ADA is compared to and a replacement is needed. The data from this study show that the CSF ADA result may potentially mislead clinicians in the diagnostic process. As such careful assessment and weighting of all the available evidence should prove more fruitful [17]. A future prospective multicentre study on a larger cohort, especially once the standardisation of CSF ADA measurement has improved, is advocated. In the meantime clinicians should be aware of the limitations of basing a diagnosis of TBM on a CSF ADA result.

## Abbreviations

ADA: Adenosine deaminase
AUC: Area under the curve
CSF: Cerebrospinal fluid
ELISA: Enzyme-linked immunosorbent assay
HIV: Human Immunodeficiency virus
IQR: Interquartile range
IQRs: Interquartile ranges
PCR: Polymerase chain reaction
ROC: Receiver operating characteristic
TB: *Mycobacterium tuberculosis* complex
TBM: Tuberculous meningitis
WHO: World Health Organisation
